# Assessment of adolescent and youth friendly services in primary healthcare facilities in two provinces in South Africa

**DOI:** 10.1186/s12913-018-3623-7

**Published:** 2018-10-22

**Authors:** Shamagonam James, Pedro T. Pisa, John Imrie, Moira P. Beery, Catherine Martin, Catherine Skosana, Sinead Delany-Moretlwe

**Affiliations:** Wits Reproductive Health and HIV Institute, School of Clinical Medicine, University of the Witwatersrand, 22 Esselen Street, Hillbrow, Johannesburg 2001 South Africa

**Keywords:** South Africa, Adolescents, Adolescent and youth health services (AYFS)

## Abstract

**Background:**

Health services for adolescents are increasingly recognised as a priority in low- and middle-income countries (LMICs). The Adolescent and Youth Friendly Service (AYFS) approach has been promoted in South Africa by the National Department of Health and partners, as a means of standardising the quality of adolescent health services in the country. The objective of this paper is to detail the evaluation of AYFS against defined standards to inform initiatives for strengthening these services.

**Methods:**

A cross-sectional assessment of AYFS was carried out in 14 healthcare facilities in a sub-district of Gauteng Province and 16 in a sub-district in North West Province, South Africa. Data on adolescent care and service management systems were collected through interviews with healthcare providers, non-clinical staff and document review. Responses were scored using a tool based on national and World Health Organisation criteria for ten AYFS standards.

**Results:**

Mean scores for the ten standards showed substantial variation across facilities in the two sub-Districts, with Gauteng Province scoring lower than the North West for 9 standards. The sub-district median for Gauteng was 38% and the North West 48%. In both provinces standards related to the general service delivery, such as Standards 4 and 5, scored above 75%. Assessment of services specifically addressing sexual, reproductive and mental health (Standard 3) showed that almost all these services were scored above 50%. Exploration of services related to psycho-social and physical assessments (Standard 8) demonstrated differences in the healthcare facilities’ management of adolescents’ presenting complaints and their comprehensive management including psycho-social status and risk profile. Additionally, none of the facilities in either sub-district was able to meet the minimum criteria for the five standards required for AYFS recognition.

**Conclusion:**

Facilities had the essential components for general service delivery in place, but adolescent-specific service provision was lacking. AYFS is a government priority, but additional support for facilities is needed to achieve the agreed standards. Meeting these standards could make a major contribution to securing adolescents’ health, especially in preventing unintended pregnancies and HIV as well as improving psycho-social management.

**Electronic supplementary material:**

The online version of this article (10.1186/s12913-018-3623-7) contains supplementary material, which is available to authorized users.

## Background

Addressing adolescent health is an important public health concern and the notion that adolescence is the healthiest period of life when compared to early childhood or adulthood is rapidly being eroded [1]. Risk behaviours in adolescence contribute substantially to the global burden of disease and it is recognised, that over the past fifty years there has been minimal improvement in adolescent health overall [1].

South Africa, like many other countries, is experiencing a health transition that also impacts on adolescents [2]. General concerns about adolescent health are augmented by concern about accidental injuries, poor mental health, substance abuse, problems related to under or over nutrition, infectious diseases and sexual reproductive health [3]. Additionally rapid urbanisation in South Africa’s cities has resulted in a growing younger urban population [4], concentrated in large metropolitan areas and in smaller medium size cities. Clearly, improving the quality of health services tailored to the needs of urban and peri-urban adolescents, has the potential to address some of the challenges resulting from rapid urbanisation, health transitions and the burden of disease associated with adolescent engagement in risk behaviours [5]. Therefore for all adolescents, particularly those using public health care services, interventions are urgently needed that address risk behaviours and their consequences including sexual and reproductive health (SRH) outcomes. To do this, a detailed understanding is needed of the factors explaining adolescent health outcomes, including the physical environment, social context, and the availability and access to social and health services [6].

In South Africa, *loveLife,* a national adolescent non-governmental organisation (NGO), in collaboration with a consortium of non-governmental organisations conceptualized, developed and implemented the National Adolescent-Friendly Clinic Initiative (NAFCI) [7]. NAFCI aimed to engage the public health sector to provide services for adolescents that address some of the barriers to service uptake through increasing the availability of non-judgemental healthcare providers, and of providing appealing, appropriately equipped and easily accessible facilities [8]. As part of this, a quality improvement approach to Adolescent and Youth Friendly Services (AYFS) was adopted, under which healthcare providers are capacitated to deliver quality adolescent services that could be measured against predetermined standards and criteria [7].

The first evaluation of AYFS programming following the South African National Department of Health’s assumption of the management of AYFS in 2006, was conducted in healthcare facilities in Mpumalanga province [9]. The evaluation found that the AYFS programme was not being implemented at the selected facilities as these facilities lacked AYFS training and space to accommodate the services [9]. This is evidenced in another study investigating young peoples’ perception of youth services in an urban setting. In this study young people reported dissatisfaction with services relating to the lack of resources, long waiting times, and poor quality of care evidenced by staff shortages, insufficient diagnostic equipment and drug stock-outs [10]. In summary they perceived the nurses to be rude and dismissive of the need for a confidential and respectful service [10].

Since these initial evaluations, there have been renewed efforts to develop and implement AYFS, but so far little is known about how successful they have been, how well facilities have aligned themselves to AYFS standards, or the extent to which AYFS is being implemented and monitored. Against this background and the need for tangible service improvements for adolescents and youth generally, and more specifically related to SRH including HIV treatment, care and management, this study undertook to retrospectively review the baseline AYFS assessments conducted at public healthcare facilities in two sub-districts, one each in Gauteng and North West Province. An assessment of the adolescent and youth data (10 to 24 years) for the quarter coinciding with the conduct of the AYFS facility appraisals, reported a headcount of 2859 in the Gauteng sub-district and 8718 for the sub-district in the North West Province. Thirty three and forty six new HIV positive adolescents and youth were identified in Gauteng and North West Province respectively. Of the HIV newly diagnosed the lost to follow up -eighteen in Gauteng and thirty-five in North West was concerning and demonstrated, among other reasons, the imperative to evaluate the baseline AYFS assessments more critically. The objective of this paper therefore, is to detail the baseline findings of a cross-sectional assessment carried out at the 30 public primary healthcare facilities –providing an overview of the quality of services available for adolescents in these two provinces in South Africa.

## Methods

Between May and July 2015, assessments of adolescent and youth friendly services were done at healthcare facilities in one sub-district in Gauteng Province and one in the North West Province. The Gauteng sub-district is a densely populated inner-city area, with almost 700,000 inhabitants, 20% of whom are young people aged 10–24 years [11]. There are 14 public healthcare facilities in the area, all of which were included in the assessment. The sub-district in the North West comprises a small-medium size city and surrounding peri-urban areas with a population of about 400,000, of which around 25% are aged 10–24 years [12]. All 16 of the sub-district’s public healthcare facilities were included. The North West Province sub-district is also one of eleven sub-districts piloting the National Health Insurance (NHI) scheme, which is planned for eventual nationwide implementation [12].

The assessment teams were drawn from the Wits Reproductive Health and HIV Institute (Wits RHI), a research institute that partners with the Department of Health (DoH) in both sub-districts, and has established relationships with the local healthcare facilities. Clinical and psycho-social Quality Improvement Advisors (QIAs) from Wits RHI provide support and mentorship to DoH staff to improve service quality. The QIA activities include providing support for facility managers, establishing AYFS teams and other mechanisms to promote AYFS at facilities, facilitating training of healthcare providers and assisting with facility self-appraisals such as that reported here (1st appraisal).

In the North West the facility self-appraisals were conducted by QIAs and facility staff, while in Gauteng it was conducted by the QIAs on their own, with facility staff only participating in interviews. The assessment of facilities’ “adolescent and youth friendliness” is guided by a self-appraisal tool, referred to as “the tool”, which provides a practical mechanism for assessing existing services for adolescents and youth from several perspectives. The tool enables identification of potential areas for improved service delivery, and further provides a standardized manner to assess several facilities. It may be used by individuals (facility managers or healthcare providers), but a team-based approach was found to be more beneficial in that it allows participation by a range of stakeholders and improves ownership of the process [13]. Teams comprised of four people (appraisal team), for example the Facility Manager, AYFS team leader and QIAs, conducted the assessments. All assessments were scheduled ahead of time, staff were informed of the objectives and the appraisal team decided on the approach to be used, for example dividing the appraisal tool into sections (standards) to be completed by individuals with the appropriate expertise related to each of the standards.

The appraisal team then conducted the assessment with minimal disruption to the clinic flow as well as being respectful of client rights. Once the appraisal team had collected all the data using the appropriate research methodologies, that included collecting data from review of clinic records and protocols; interviews with facility managers, healthcare providers and non-clinical staff; observation of client-provider interactions; and inventories of the healthcare facility and its immediate surroundings, a meeting was convened to collate the findings and reach consensus on areas of discrepancy. All data collected was verified with the Facility Managers.

The appraisal teams were trained to use the standardised tool developed by the National Department of Health and *loveLife* [13] These standards were developed by Wits RHI as part of NAFCI, following a lengthy process of consultations with professionals and youth, and informed the development of the World Health Organisation (WHO) recommended standards for the field [8], which have recently been updated [8]. The tool includes more than 40 performance criteria (Additional file 1), which are organised into ten Standards of services and management systems [14]. The ten Standards are:

1. **Management System Support for the effective provision of adolescent and youth friendly health services**
2. Policies and processes that support the rights of adolescents
3. **Appropriate adolescent health services are available and accessible**
4. **The clinic has a physical environment conducive to the provision of adolescent friendly health services**
5. The clinic has adequate drugs, supplies and equipment necessary to provide the essential service package for youth-friendly healthcare
6. **Provision of relevant information, education and communication (IEC) promoting behaviour change and consistent with the YFS essential service package**
7. Systems in place to train and develop staff to provide effective adolescent-friendly health services
8. Adolescents receive adequate psycho-social and physical assessments
9. Adolescents receive individualised care based on standard case management guidelines/protocols
10. **The clinic provides continuity of care for adolescents: proper referral systems are in place**

Achievement of Standards 1, 3, 4, 6 and 10 is the minimum requirement for AYFS recognition.

As sexual and reproductive health (SRH) is a particularly important aspect of adolescent health, and in many instances the entry point to health services for adolescents, a sub-set of five of the ten criteria from Standard 3 that relate to SRH, including violence/abuse and mental health are reported in detail.

Team leaders were responsible for scoring the standards according to how each of the statements in the criterion were answered (Yes/No). To achieve uniformity, the following method of scoring the criteria was adhered to: all statements answered “Yes” implied the criterion was met and awarded 2 points; a mix of “Yes” and “No” to statements in a criterion where the “Yes” comprised more than 50%, implied the criterion was partially met and awarded 1 point. All statements answered “No” implied the criterion was not met and awarded 0 points. A mix of “Yes” and “No” where the “No” was equal to or more than 50% implied the criterion was not met and awarded 0 points. All the criteria in each standard were scored to obtain the total points for the standard. The maximum points for a standard is obtained by adding the number of criteria in the standard and multiplying the result by 2. The overall score for the standard is determined by taking the score for the standard and dividing it by the possible total for the standard, then multiplying by 100 to obtain a percentage score for the standard. All scores were then verified independently by the researcher. A high degree of accuracy and consistency in the scoring was observed. All assessments were finally discussed with facility staff through feedback reports and both hard and digital copies handed over for future reference, development of action plans and the conduct of subsequent appraisals. Median scores for the standards and the sub-districts were calculated. Final Scores were entered into Microsoft Access 2013 and exported to Microsoft Excel 2013 for descriptive analysis.

Approval to use the AYFS program data for the purpose of this manuscript was granted retrospectively by the University of the Witwatersrand Human Research Ethics Committee (certificate number: M160227), and the City of Johannesburg Health District and the North West Province Research Committees. Informed consent was not sought from individual facility managers and healthcare providers as this was routinely collected facility programme assessments. Permission to publish the analysed assessments was requested retrospectively as all assessments conducted occurred prior to the sitting of the ethics review committee [15]. Funding for Wits RHI Adolescent Innovations Project was through USAID Southern Africa (Cooperative Agreement No.AID-674- A12–00032: HIV Innovation for Improved Patient Outcomes for Priority Populations-Adolescents). The funding body was not involved in any research processes -study design, data collection, and/or data analysis.

## Results

The overall average score across the ten standards for each healthcare facility, the median scores and ranges for the standards and the sub-districts are presented in Tables 1 and 2. The sub-district median score and range across the facilities for Gauteng was 37% (27–49%) and the North West 48% (36–75%). The standard median scores for the facilities in Gauteng were all lower than that of the North West with the exception of Standard 4 (83%) that equalled the North West.

**Table 1:** Standard Scores for Facilities in Gauteng Sub-District (*n* = 14)

	**Std 1** **Management System Support (%)**	Std 2 Policies and Processes (%)	**Std 3** **Appropriate Adolescent Health Services (%)**	**Std 4** **Physical Environment (%)**	Std 5 Adequate Drug, Supplies and Equipment (%)	**Std 6** **IEC Provision (%)**	Std 7 Staff Training (%)	Std 8 Adequate psycho-social and Physical Assessment (%)	Std 9 Individual Care (%)	**Std 10)** **Continuity of Care (%)**	**Overall Facility Average (%)**
Facility 1	**0**	10	**35**	**83**	83	**0**	17	0	17	**25**	**27**
Facility 2	**0**	30	**40**	**83**	67	**0**	17	38	0	**0**	**28**
Facility 3	**0**	20	**35**	**83**	100	**0**	0	13	17	**25**	**29**
Facility 4	**0**	40	**40**	**83**	100	**0**	17	38	0	**0**	**32**
Facility 5	**0**	30	**40**	**67**	50	**35**	0	63	33	**0**	**32**
Facility 6	**0**	20	**45**	**83**	83	**0**	0	38	50	**25**	**34**
Facility 7	**0**	10	**30**	**83**	100	**0**	17	38	50	**25**	**35**
Facility 8	**8**	30	**40**	**83**	83	**0**	0	75	50	**25**	**39**
Facility 9	**0**	30	**45**	**83**	67	**0**	17	50	83	**25**	**40**
Facility 10	**0**	30	**55**	**67**	67	**0**	33	38	83	**50**	**42**
Facility 11	**17**	30	**45**	**67**	67	**17**	33	100	33	**25**	**43**
Facility 12	**0**	30	**45**	**67**	83	**17**	17	100	83	**0**	**44**
Facility 13	**8**	20	**55**	**67**	67	**67**	50	75	83	**0**	**49**
Facility 14	**8**	30	**50**	**67**	50	**0**	100	100	50	**39**	**49**
**Std Median (Range)**	**0 (0–17)**	30 (10–40)	**43 (35–55)**	**83 (67–83)**	75 (50–100)	**0 (0–67)**	17 (0–100)	44 (0–100)	50 (0–83)	**25 (0–50)**	
**Sub-District Median** **(Range)**											**37 (27–49)**

Overall facility average is average of all 10 Standards for the facility. Std Median is median of the individual standard across the facilities in the sub-district. Sub-District Median is the median of the facilities across the sub-district. Standard fully met: 100%; Standard partially met: ≥50–-99%; Standard not met: < <50%. Std Standard

**Bold**: Minimum Standards required for AYFS Recognition (Standards 1, 3, 4, 6 and 10)

**Table 2:** Standard Scores for Facilities in North West Sub-District (*n* = 16)

	**Std 1** **Management System Support (%)**	Std 2 Policies and Processes (%)	**Std 3** **Appropriate Adolescent Health Services (%)**	**Std 4** **Physical Environment (%)**	Std 5 Adequate Drug, Supplies and Equipment (%)	**Std 6** **IEC Provision (%)**	Std 7 Staff Training (%)	Std 8 Adequate psycho-social and Physical Assessments (%)	Std 9 Individual Care (%)	**Std 10** **Continuity** **Of Care** **(%)**	**Overall Facility Average (%)**
Facility 1	**0**	30	**45**	**66**	83	**0**	16	50	17	**50**	**36**
Facility 2	**8**	30	**50**	**67**	67	**0**	17	75	33	**50**	**40**
Facility 3	**0**	0	**60**	**83**	83	**0**	50	38	100	**0**	**41**
Facility 4	**0**	40	**45**	**67**	67	**0**	0	63	33	**50**	**41**
Facility 5	**8**	20	**50**	**67**	83	**0**	17	75	50	**50**	**42**
Facility 6	**8**	40	**55**	**100**	100	**0**	17	50	66	**25**	**46**
Facility 7	**8**	20	**60**	**83**	83	**0**	33	38	83	**50**	**46**
Facility 8	**0**	60	**50**	**83**	100	**0**	33	25	100	**25**	**48**
Facility 9	**8**	50	**45**	**100**	100	**0**	17	25	83	**50**	**48**
Facility 10	**58**	40	**50**	**66**	83	**0**	50	75	50	**50**	**52**
Facility 11	**33**	50	**55**	**83**	83	**0**	50	75	50	**50**	**53**
Facility 12	**8**	40	**75**	**100**	100	**0**	17	50	100	**50**	**54**
Facility 13	**33**	50	**65**	**83**	83	**0**	33	100	67	**50**	**56**
Facility 14	**8**	40	**55**	**83**	83	**0**	17	100	100	**75**	**56**
Facility 15	**8**	70	**60**	**83**	83	**0**	67	63	83	**50**	**57**
Facility 16	**67**	60	**85**	**83**	100	**0**	100	75	100	**75**	**75**
**Std Median (Range)**	**8 (0–67)**	40 (0–70)	**55 (45–85)**	**83 (66–100)**	83 (67–100)	**0**	25(16–100)	63 (38–100)	75 (17–100)	**50 (0–75)**	
**Sub-District Median (Range)**											**48 (36–75**)

Overall facility average is average of all 10 Standards for the facility. Std Median is median of the individual standard across the facilities in the sub-district. Sub-District Median is the median of the facilities across the sub-district. Standard fully met: 100%; Standard partially met: ≥50–-99%; Standard not met: < <50%. Std Standard

**Bold**: Minimum Standards required for AYFS Recognition (Standards 1, 3, 4, 6 and 10)

### Standards required for AYFS recognition (1, 3, 4, 6 and 10)

Tables 1 and 2 also highlight the scores (bold) for the five standards that need to be achieved for AYFS recognition for each of the facilities in both provinces. The facility scores for Standard 1 that measure management systems that facilitate AYFS implementation were low in all facilities with the exception of two facilities in the North West that partially met the criteria with scores of 58% and 67% respectively. The standards measuring adolescent specific services (Standards 6 and 10) generally scored low with the exception of two facilities in the North West achieving partially met scores of 75% for Standard 10 that measured continuity of care. The scores for Standard 3 that included measures for specific initiatives to make services accessible and available to adolescents were generally higher for individual facilities in the North West than Gauteng with the median standard score in the North West (55%) being higher than Gauteng (43%). More promising results were reported for Standard 4 that measured the physical environment for safety and infection control measures. More than half the facilities in both provinces had scores equal to or higher than 83%.

In both provinces none of the facilities achieved all five standards, a criteria for recognition as an AYFS facility, aside from one facility in the North West that partially met the criteria for all the five standards except Standard 6.

### Assessment standards other than those required for AYFS recognition (2, 5, 7, 8 and 9)

Assessment of the five standards that were not required for AYFS recognition showed a similar pattern to the standards required for AYFS recognition. The standards related to management systems (Standard 2) and AYFS specific activities (Standards 7, 8 and 9) scored lower than the standard that was related to general healthcare delivery (Standard 5). The median scores for all standards in this category in the North West were higher than in Gauteng. With the exception of two facilities in Gauteng, all other facilities in both provinces either fully or partially met the criteria for Standard 5 that assessed the adequate provision of drugs, supplies and equipment. Majority of the facilities in the North West either fully or partially met the criteria for providing adolescents with services that met their needs in an adequate and individualised manner (Standards 8 and 9).

### Clinical services for sexual, reproductive and mental health

**Fig. 1:**
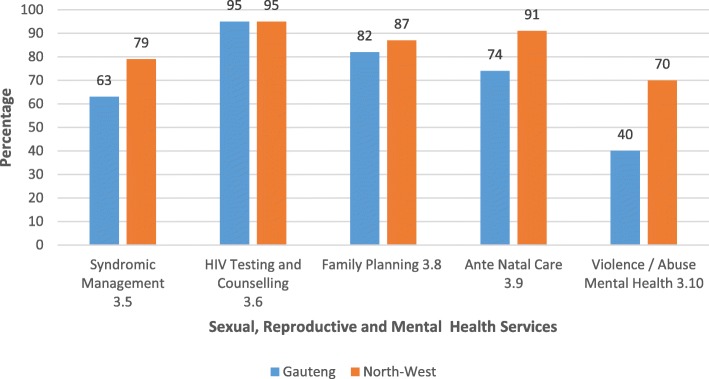
Standard 3 – Adolescent Sexual Reproductive and Mental Health Services (Criteria 3.5, 3.6, 3.8, 3.9, and 3.10)

The availability of five clinical services that are essential components of SRH services for adolescents are shown in Fig. 1. These services are syndromic management of sexually transmitted infections (STIs); HIV counselling and testing; family planning services, antenatal care (ANC), and management and referral of violence, abuse and mental health issues. In both sub-districts, the criteria for these essential SRH services were at least partially met in nearly all facilities. The higher scores achieved were for HIV counselling and testing and family planning services indicating widespread availability of these services. Facilities in the North West scored higher than those in Gauteng for all of these essential clinical services, aside from HIV counselling and testing where both scored 95%. The score for measures addressing violence and abuse in Gauteng was low (40%).

### Physical and psycho-social assessment of adolescents

**Fig. 2:**
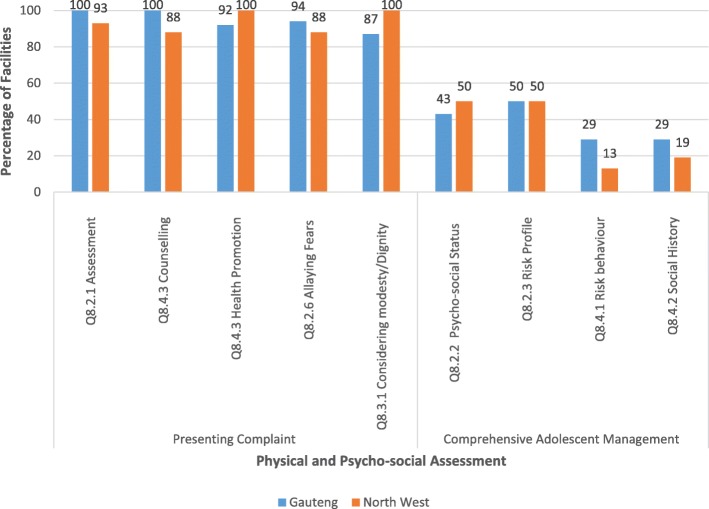
Standard 8 – Adolescents Receive Adequate Psycho-social and Physical Assessment (Criteria 8.2, 8.3 and 8.4)

An exploration of the criteria related to the adequate physical and psycho-social assessments of adolescents is shown in Fig. 2. The individual statements in the criteria are broadly separated into assessments related to the presenting problem and those related to overall comprehensive care that takes into account the social background of adolescents as well as their risk for HIV, STIs and unintended pregnancies. In both provinces majority of the facilities, more than 85%, adequately addressed adolescents for their presenting problem in terms of the physical examination, providing health promotion and considering the patients’ needs (fears and dignity). Fifty percent and less of these facilities indicated considering the adolescents’ psycho-social status and risk profile and conducting the periodic behaviour-risk assessment and social history.

Overall, the higher scores on Standards 3, 4, 5, 8 and 9 showed that substantial number of facilities had the necessary infrastructure and essential components in place to deliver basic services.

## Discussion

The AYFS assessments in public healthcare facilities in two South African health sub-districts found that overall facilities did not meet the criteria for youth-friendly service provision, as set out by the National Department of Health guidelines [16]. In both sub-districts, facilities did not satisfy the criteria for the five minimum standards required for AYFS recognition, although performance in relation to some specific clinical service provision criteria was encouraging. The large variation in scores between facilities is a particularly notable finding as it signals the presence of high levels of inequities in access. Also, given the high levels of poly-victimisation, violence and abuse in Gauteng [17], it is cause for concern that services related to violence/abuse and mental health were not met.

In both sub-districts, two standards in particular, those pertaining to the clinic environment (Standard 4) and the presence of drugs, supplies and equipment (Standard 5), scored higher than other measures. These standards largely reflect the general requirements for a functioning public healthcare facility, rather than aspects specific to adolescent services. Facilities fared poorly in the measures that were more specific to adolescent services, for example supportive management (Standard 1), policy and processes (Standard 2) and information, education and communication strategies (Standard 6). This was true for both areas, though overall the North West facilities scored higher than those in Gauteng. It is possible that the higher scores in the North West reflect the advances that have taken place with the prioritized strengthening of service delivery platforms in the NHI pilot sites [18]. Included in the strengthening of health system activities was AYFS quality improvement initiatives supported by the district and support partners. These activities initially focussed on AYFS training for healthcare providers that included training related to improving the clinical and psycho-social care and management of adolescents, conducting support groups and instituting more user friendly times and days for adolescents to access care and treatment. The QIAs supporting the healthcare facilities were instrumental in these processes and evidence of improved healthcare provider engagement and attitudes as well as patient satisfaction were noted in an evaluation of the I ACT (Integrated Access to Care and Treatment) support group activity, despite on-going logistic constraints [19].

Considering QIA and technical support to all facilities was constrained by the number of staff available, facilities were initially prioritised for targeted technical support according to a pre-determined criteria that included, facility performance and number of adolescents in the clinic catchment area including adolescents identified with HIV. Quality improvement efforts were initiated after reviewing the facilities’ contextual factors relevant to operations and sustainability, for example, facility manager support; availability of resources including AYFS-trained nurses; Ward Based Outreach Teams (WBOTS); School Health Nurses and loveLife collaborators. Remaining facilities were systematically recruited into the quality improvement initiative. However, contingency plans for unanticipated delays and efforts towards building sustainability into the quality improvement process could benefit from formulating a supportive intervention strategy between high performing and low performing facilities. Linking the facilities may be initiated by the Quality Improvement Advisors who are familiar with the sub-districts’ needs, healthcare facilities and management structures.

The AYFS assessment tool, while guiding the evaluation of facilities also provides a benchmark for developing and implementing quality improvement initiatives and for standardisation of adolescent service provision across facilities. The detail provided in the individual criteria is important for guiding the quality improvement planning for individual facilities, while the standard scores gives a higher-level overview of performance. Of particular note, averaging facilities’ criteria scores to derive an overall standard score can mask important areas of strength and weakness in facilities. This is clearly demonstrated in the comparison of low overall scores for Standard 3 versus the relatively high individual scores for provision of some essential clinical SRH and mental health services. Also, of significant note is the detail that can be gleaned from assessing the responses to individual statements in the criteria. The deeper exploration of Standard 8, statements related to the provision of adequate physical and psych-social care of adolescents demonstrated that facilities were adequately prepared to manage adolescents’ physical or presenting complaint to a greater extent than they were able to provide services that contribute to comprehensive care and management. The latter services were specifically related to adolescent assessment of psycho-social status and risk for HIV, STIs and unintended pregnancy.

This paper offers important data reflecting the general lack of adolescent specific services which can inform the implementation of AYFS nationally as envisaged by the National Department of Health and the South African National AIDS Council (SANAC). Adolescent health and delivery of AYFS is a priority of the South African government. In 2016, the National Department of Health launched the National Campaign for Adolescent Girls and Young Women (www.sheconqurerssa.co.za). The campaign aims to empower young women and girls through targeted approaches that increase economic support and reduce HIV, teenage pregnancies, school dropout and gender based violence [20]. The comprehensive package of interventions that includes biomedical prevention interventions for young women and adolescent girls is envisaged to use AYFS as an approach to promote access to SRH services and information [20]. The recommended operational strategy is the creation of single service point of delivery models that integrate HIV and SRH services –Youth Zones. Youth Zones aim to meet the practical and psychosocial needs of young people by including operating times that are suitable to them and providing services that are private and by staff who are non-judgemental [21].

Data on AYFS in South Africa is limited, and there are still only a few international studies that demonstrate outside of a research context the impact of improving the friendliness of services on adolescent health and service uptake. A study in Zambia that implemented youth friendly services projects in selected facilities found increased satisfaction among young patients with the services they received and nurses more supportive of providing SRH services to young people [22]. The same study also demonstrated the importance of contextual factors by showing that social and community level factors were equal, if not more influential than health system factors, in determining youth service uptake [22].

The application of the AYFS assessment tool shows how facilities can identify areas that are working well and areas that require strengthening. Encouragingly, the appraisal of criteria related to provision of HIV testing and counselling and family planning services for both sub-districts scored at the higher end of the scale. These infrastructural capabilities indicate the potential of the sub-districts to accommodate the anticipated rise in demand for SRH services, as a result of increased urbanisation and socio-economic changes in the country [23]. Such service demands may be heightened for a sub-district like that in Gauteng which is characterised by a dense inner-city population. Gaps in the provision of pregnancy services (ANC), STI screening and treatment, and management of violence, abuse and mental health issues not only have critical implications for individual adolescents, but also for public health and prevention in general. Facilities need to prioritise making services friendly for adolescents with interventions like opening hours that allow young people to come outside of school hours, and staff who are friendly to adolescent patients [24]. Overall such services need to be delivered in a manner that is available to adolescents, but also acceptable and accessible [8].

Some study limitations warrant mention. The measures of access to SRH services included HIV testing and counselling, but not other key components of HIV services for adolescents, are a major gap in our data. Also, an assessment of adolescent perceptions would have allowed for a more comprehensive evaluation of the services. Improved understanding of adolescent felt needs and perceptions of the healthcare system is important to ensure public healthcare facilities attract adolescents, meet their needs and retain them in care [25]. Additionally, an assessment of the actual barriers to adolescent service provision from the healthcare providers may help to strengthen proposed on-going initiatives. Lastly, as this analysis does not lend itself to explaining the causes of the overall poor assessment outcomes, post-assessment engagement through feedback processes with facility staff is crucial. Such engagement is intended to identify gaps, develop action plans and discuss recommendations to overcome some of the weaknesses identified. Repeat assessments of the facilities should be conducted to evaluate whether and how this assistance affects AYFS scores.

## Conclusion

This assessment showed that all facilities had the essential components for service delivery in place - basic infrastructure, SRH services and essential drugs and equipment. It also suggests that South Africa has the necessary tools to guide both implementation and assessment of the country’s policy and plan for AYFS [16]. The findings however, showed disconnect between the implementation policy and facility-level performance. Concerted efforts to develop systems that apply the policy and principles of AYFS in the functioning of facilities are undeniably needed. These initiatives will not only benefit adolescents but the health system overall, as the principles for implementation of the NHI are in step with those of AYFS – effectiveness, affordability, appropriateness, equity and efficiency. Developing and implementing a comprehensive AYFS package inclusive of community and outreach activities also aligns to the overhaul of the national primary healthcare efforts [26].

## Additional file

Additional file 1: YFS Self-Appraisal Framework. Adolescent and Youth Friendly Services assessment tool for use at healthcare facilities. (DOC 2674 kb)

## Abbreviations

ANC: Antenatal Care
AYFS: Adolescent and Youth Friendly Services
DoH: Department of Health
HIV: Human Immunodeficiency Virus
LMICs: Low-and Middle Income Countries
NAFCI: National Adolescent Friendly Clinic Initiative
NGO: Non-governmental Organisation
NHI: National Health Insurance
QIAs: Quality Improvement Advisors
SANAC: South African National AIDS Council
SRH: Sexual and Reproductive Health
STIs: Sexually Transmitted Infections
WHO: World Health Organisation
Wits RHI: Wits Reproductive Health and HIV Institute
