# Supplementary material for: Assessment of adolescent and youth friendly services in primary healthcare facilities in two provinces in South Africa
Source: BMC Health Serv Res. 2018 Oct 22;18:809. doi: 10.1186/s12913-018-3623-7 (PMC6198437; doi:10.1186/s12913-018-3623-7)
Supplement: Supplementary file 1 — YFS Self-Appraisal Framework. Adolescent and Youth Friendly Services assessment tool for use at healthcare facilities. (DOC 2674 kb) [file 12913_2018_3623_MOESM1_ESM.doc]

13

**Tool # 1**

**YFS SELF-APPRAISAL FRAMEWO****RK**

**STANDARD 1**

**Management systems are in place to support the effective provision of youth-**

**friendly health services.**

**INTENT**

**The management systems that are place ensure that health services effectively**

**meet the needs of adolescent clients. The clinic’s service plan is based on**

**information obtained about young people in the community through the community**

**profile, adolescent needs assessments, and data from the clinic’s health**

**information system.**

**CRITERION 1.1**

**Data are collected to determine adolescent’s health needs.**

**Questions 1st 2nd 3rd 4th**

**appraisal appraisal appraisal appraisal**

Has an adolescent health profile been conducted within

the last 2 years?

1.1.1

Have young people’s sexual and reproductive health

challenges been clearly identified in the profile?

1.1.2

Has an assessment of needs and preferences of young

people for SRH services been conducted within the past

year (e.g. needs assessment, focus group discussions,

surveys, client exit interviews, interviews with

adolescent/youth key informants)?

1.1.3

Are the findings of the needs assessments and

adolescent health profile communicated to staff?

1.1.4

15

Is information on young people’s uptake of the YFS

service package being collected by age and by gender

of the client (e.g. M/F; 10-14, 15-19, 20-24 year olds)?

1.1.5

Is the data on young people’s service utilization

complied and analyzed on a monthly basis to detect

trends in young people’s health needs?

1.1.6

Are YFS’ service trends regularly discussed with staff,

volunteers and members of the community health

committee?

1.1.7

**CRITERION 1.2**

**The clinic has a service plan that addresses the need for adolescent health services**

**and a process to implement the plan**

**Questions 1st 2nd 3rd 4th**

**appraisal appraisal appraisal appraisal**

Has a YFS service plan been developed?

1.2.1

Does the service plan reflect the health priorities

outlined in the adolescent health profile and the needs

identified by research with young people?

1.2.2

Do all staff and volunteers know the contents of the

YFS service plan?

1.2.3

Is the YFS service plan posted (with times) in an area

where young people are likely to see it (e.g. reception

area, chillroom, etc.)?

1.2.4

Are all of the services described in the YFS service

plan currently being provided?

1.2.5

**CRITERION 1.3**

**Staff receive support and supervision on an on-going basis.**

**Questions 1st 2nd 3rd 4th**

16

**appraisal appraisal appraisal appraisal**

Do staff feel that they receive adequate support from

on-site managers / supervisors to deliver YFS?

1.3.1

Do staff feel that they receive adequate support from

off-site managers / supervisors to deliver YFS?

1.3.2

Do managers / supervisors regularly encourage

improvements in the quality of young people’s care?

1.3.3

Do managers / supervisors regularly assess staff

competency and performance in relation to YFS?

1.3.4

Do managers / supervisors provide constructive

feedback on ways to improve the quality of services for

young people?

1.3.5

Do managers / supervisors ensure that staff are

assigned responsibility and given sufficient time to carry

out the following functions:

Participate in YFS service improvement projects

Give health talks to adolescent and youth clients

Participate in other activities for young people (at

the clinic or in the community)

Conduct community relations activities, including

joint activities with other youth serving

organizations

Maintain up-to-date YFS monitoring records

Share information and lessons about YFS

1.3.6

Do managers / supervisors oversee the progress of the

clinic’s YFS’ service improvement efforts and

programme activities to ensure that staff are staying on

track and to provide guidance?	1.3.7

Is team spirit fostered in the clinic to support YFS?

1.3.8

**CRITERION 1.4**

**The clinic has a regular process for improving the quality of services for**

**adolescents.**

**Questions 1st 2nd 3rd 4th**

**appraisal appraisal appraisal**
**appraisal**

17

Have all categories of staff been oriented in the YFS

programme?

1.4.1

Has a clear structure for YFS been mapped out in a YFS

Programme Management Plan?

1.4.2

Has the clinic’s commitment to YFS been formally

declared in a YFS Charter?

1.4.3

Has a YFS Team been established?

1.4.4

Does the YFS Team have a broad representation (e.g.

all professional and non-professional backgrounds,

youth and community representation)?

1.4.5

Does the YFS Team approach YFS’ service

improvement process systematically by:

Meeting regularly (e.g. once a month)

Setting meeting agendas

Keeping an attendance register

Taking minutes

Establishing project teams to address service gaps

Documenting project action plans

Documenting project outcomes and outcomes of

YFS’ other service improvement efforts

1.4.6

Does the YFS Team regularly share their meeting

minutes, project plans or project results with staff,

volunteers and other YFS stakeholders (e.g. CHC, local

schools, etc.)?

1.4.7

Does the YFS Team ensure that staff and volunteers

have the information necessary to actively participate in

the projects and activities set forth in YFS’ plans?

1.4.8

Are educational sessions for YFS Team members and

other members of staff about YFS’ service improvement

methods and tools taking place on a regular basis?

1.4.9

Do the YFS Coordinator and YFS Team Leader actively

coach/mentor the YFS Team and other members of staff

in applying YFS’ methods?

1..4.10

Is there a mechanism in place to ensure that all

staff/volunteers are involved with some aspects of YFS’

decision making, even when they are not core members

of the YFS Team (e.g.
prioritization of future service

18

improvement goals)?

1.4.11

Are the results from the analysis’ of YFS’ service

utilization trends being used to inform programme

planning?

1.4.12

Are the results from YFS self-appraisals being used to

inform programme planning?

1.4.13

Are young people’s suggestions/ complaints about

services being used to inform programme planning (e.g.

results from client satisfaction surveys, client exit

interviews, suggestion box, etc.)?

1.4.14

Are the results from the adolescent needs assessments

and adolescent health profile being used to inform

programme planning?

1.4.15

Is there a mechanism in place to formally recognize

team and individual efforts in the delivery of YFS?

1.4.16

Are YFS’ successes and testimonials well showcased

throughout the clinic?

1.4.17

**CRITERION 1.5**

**The clinic has a system to assure adolescent and community participation in the**

**planning and provision of services**

**Questions 1st 2nd 3rd 4th**

**appraisal appraisal appraisal appraisal**

Does the YFS Team include at least 2 youth

representatives?

1.5.1

Are young people involved in providing educational

programmes, peer counselling or other youth activities

at the clinic?

1.5.2

Does the Community Health Committee play an active

role in supporting YFS?

1.5.3

19

Are young people represented on the Community Health

Committee (e.g. at least 2 youth members)?

1.5.4

Are outside speakers invited to present on topics related

to YFS?

1.5.5

Have links been established with other facilities

implementing YFS to share experiences and resources

and/or to jointly carry out selected activities?

1.5.6

Have links been established with local youth

stakeholders to share experiences and resources and/or

to jointly carry out selected activities (e.g. community

elders/leaders, youth leaders, schools, youth

organizations, parents, etc.)?	1.5.7

**CRITERION 1.6**

**The clinic has an adequate client record system**

**Questions 1st 2nd 3rd 4th**

**appraisal appraisal appraisal appraisal**

Are young people’s records completed with the

following information:

Age

Sex

Identification number

Findings of physical examination

Sexual history

Social history

Behaviour Risk Assessment

Relevant tests or lab ordered with results

Diagnosis

Counselling

Treatment

Appropriate referral

Follow-up/return date	1.6.1

Is there a record filing system in place where young

people’s records can easily be located?
1.6.2

20

Is there a process in place for identifying and

following up on high-risk adolescent /youth clients

(e.g. a log book)?
1.6.3

21

**STANDARD 2**

**The clinic has policies and processes that support the rights of adolescents.**

**INTENT**

**There are policies and processes to ensure that the rights of adolescents are**

**known and respected by all clinic staff. Services are provided taking into account**

**the rights of adolescents.**

**CRITERION 2.1**

**The clinic has a copy of the National Youth and Adolescent Health Policy**

**Guidelines**

**Questions 1st 2nd 3rd 4th**

**appraisal appraisal appraisal appraisal**

Is a copy of the National Youth and Adolescent

Health Policy kept in a place that is well known and

easily accessible to staff?

2.1.1

Are staff members able to articulate the main points

of the National Youth and Adolescent Health Policy?

2.1.2

Is an overview of the National Youth and Adolescent

Health Policy included as part of the orientation of

new staff members?

2.1.3

Is the National Youth and Adolescent Health Policy

reviewed at least once a year through staff meetings

and/or during in-service training?

2.1.4

**CRITERION 2.2**

**Clinic staff know the adolescent sexual and reproductive health (SRH) rights**

**Questions 1st 2nd 3rd 4th**

**appraisal appraisal appraisal appraisal**

Are all staff members well informed of young

people’s sexual and reproductive health (SRH)

rights?
2.2.1

22

**CRITERION 2.4**

**Clinic staff provide services taking into account the rights of adolescents**

Are young people’s sexual and reproductive health

(SRH) rights reviewed during staff orientation?

2.2.2

Do all staff know at least five of these rights?

2.2.3

Is there a mechanism in place to review how each

of these rights are being met by the clinic?

2.2.4

**CRITERION 2.3**

**The clinic proactively promotes the SRH rights and responsibilities of adolescents**

**Questions 1st 2nd 3rd 4th**

**appraisal appraisal appraisal appraisal**

Does the clinic have posters on display outlining young

people’s sexual and reproductive health (SRH) rights

and responsibilities?

2.3.1

Are these posters displayed in the appropriate

language(s) for the surrounding community?

2.3.2

Are pamphlets describing SRH rights and

responsibilities readily available for young people to

take home?

2.3.3

Do healthcare providers regularly speak with young

people about their SRH rights and responsibilities?

2.3.4

Do healthcare providers and/or youth volunteers

include an overview of young people’s SRH rights and

responsibilities when conducting clinic health talks?

2.3.5

Do healthcare providers and/or youth volunteers

include an overview of young people’s SRH rights and

responsibilities when conducting community outreach

activities?
2.3.6

23

**Questions 1st 2nd 3rd 4th**

**appraisal appraisal appraisal appraisal**

Are all staff members known to young people by their

names (e.g. staff wear name tags and introduce

themselves by name to their clients)?	2.4.1

Do all staff members treat young people with a

respectful and non-judgmental attitude?

2.4.2

Are all services available to young people regardless

of their age, sex, whether or not they have parental

consent or their marital status?	2.4.3

Do staff members understand the needs and

vulnerabilities associated with different groups of

young people. For example:

Males versus females

Young people infected or affected by HIV or AIDS

Young people who are gay, lesbian or bisexual

Disabled young people

Victims of sexual or domestic violence

Perpetrators of sexual or domestic violence

Different social and ethic groups

Young people with mental health problems (e.g.

depression, eating disorders, addictions, etc.)

Orphans

Young people who are homeless

Sex workers	2.4.4

Does the clinic properly accommodate young people

with special needs (e.g. wheelchair bound, deaf,

blind)?	2.4.5

**CRITERION 2.5**

**Providers and staff maintain confidentiality of adolescent clients**

**Questions 1st 2nd 3rd 4th**

**appraisal appraisal appraisal appraisal**

Do staff members avoid discussing young people’s

problems with parents or other people in the

community?	2.5.1

Is the importance of maintaining patient confidentiality

regularly discussed in staff meetings?

2.5.2

24

Is the importance of maintaining patient confidentiality

discussed during staff orientation?

2.5.3

Are measures taken to ensure that consultations,

counselling sessions and/or examinations are not

interrupted?

2.5.4

When staff need to discuss a client’s case with other

staff, do they respect the client’s confidentiality by

speaking in a private area so that the conversation

cannot be overheard?	2.5.5

Does the clinic ensure that young people do not have

to verbally announce which services they have come

for in public areas (e.g. the waiting area or the

corridor)?	2.5.6

When a third party is present during a consultation,

examination or procedure, do staff explain the person’s

presence and ask their client’s authorization for it?

2.5.7

In presentations with groups of young people, does the

facilitator request that the information shared within the

group be kept confidential?

2.5.8

Are client records stored in such a way that the

confidentiality of the client is maintained?

2.5.9

Are staff careful not to leave client records unattended

on desktops or in other non-secure locations?

2.5.10

Does the clinic have a system in place to ensure the

confidentiality of HIV-test results?
2.5.11

25

**STANDARD 3**

**Appropriate adolescent health services are available and accessible.**

**INTENT**

**Adolescents are aware of the health services available to them. During the official**

**hours of operation, every effort is made to accommodate the needs of adolescents.**

**Where possible, specific times are allocated for the provision of adolescent**

**services. Adolescents are welcomed in the clinic and provided with the full range**

**of services. The essential service package for youth-friendly clinics is provided.**

**There is a mechanism in place to solicit community support for adolescent health**

**services.**

**CRITERION 3.1**

**The scheduling, location, and scope of adolescent services provided by the clinic**

**are clearly visible and communicated to the community**

**Questions 1st 2nd 3rd 4th**

**appraisal appraisal appraisal appraisal**

Does the clinic have a sign outside that shows:

The name of the clinic

The days of the week the clinic is open

The hours the clinic is open

An indication that young people are welcome

(or that this is a “Youth Friendly” clinic)	3.1.1

Are clinic signs posted so that they assist

clients to find the clinic from the main roads?

3.1.2

Are signs used within the clinic to ensure that

service points are easily identifiable?

3.1.3

**CRITERION 3.2**

**The clinic actively promotes adolescent health services within the community.**

**Questions 1st 2nd 3rd 4th**

**appraisal appraisal appraisal**
**appraisal**

26

Have formal service partnerships been established with

community youth organizations or other relevant

sectors (e.g. schools, NGOs, faith based organizations,

police, social services)?	3.2.1

Are activities regularly conducted to educate the wider

community (including young people) about the clinic’s

YFS initiative and other topics related to young people’s

sexual and reproductive health?

3.2.2

Do the following places where young people gather

display signs that indicate the availability of your clinic’s

‘youth friendly services’:

Schools

Community centres

Recreation centres

Local youth serving organizations

Local businesses	3.2.3

Are activities conducted to encourage parents to

support their children’s involvement with YFS?

3.2.4

Are notices of YFS’ events and educational

programmes appropriately advertised?

3.2.5

Does the clinic work with local media to disseminate

information about YFS (e.g. newspapers, radio shows,

local newsletters)?

3.2.6

**CRITERION 3.3**

**Services are provided within time frames convenient for adolescents.**

**Questions 1st 2nd 3rd 4th**

**appraisal appraisal appraisal appraisal**

During clinic hours, are all services available to young

people regardless of:

The day of the week

The time of day

The availability of a clinic card

3.3.1

Have staff made an effort to provide services based on

the times that are most convenient to adolescents (e.g.

after school, weekends)?
3.3.2

27

Has a client flow analysis been conducted to assess

waiting times?

3.3.3

Are services provided within waiting times that are

acceptable to young people?

3.3.4

Are young people’s waiting times unnecessarily

lengthened by waiting for a doctor to do something

that a nurse or other healthcare provider could do?

3.3.5

Are there enough staff available to ‘fast-track’

adolescent/youth clients when the clinic is the most

busy?	3.3.6

**CRITERION 3.4**

**All staff including reception, clerical, and housekeeping staff, are able to assist**

**youth to access care in an informed, non-judgmental manner.**

**Questions 1st 2nd 3rd 4th**

**appraisal appraisal appraisal appraisal**

Are all staff members aware that the clinic

provides youth friendly services?

3.4.1

Do all staff members know when and where

each of the services are provided?

3.4.2

Are all staff members able to direct young

people to the appropriate service areas?

3.4.3

**CRITERION 3.5**

**Syndromic management of sexually transmitted infections is provided.**

**Questions 1st 2nd 3rd 4th**

**appraisal appraisal appraisal appraisal**

Is a formal review of the quality of STI care done at least

once a year by a supervisor using the DISCA (District

STI Quality of Care Assessment) instrument?
3.5.1

28

Have all professional nursing staff been trained in the

syndromic management of STIs?

3.5.2

Are appropriate flow charts available on the wall or desk

in consultation and examination rooms (e.g. showing the

up-to-date protocols for STI management)?

3.5.3

Is there a mechanism in place to ensure that staff are

diagnosing and treating STIs according to current

protocols and treatment guidelines?

3.5.4

Are young people’s dignity and fears respected during

examinations (e.g. when they have their skin, mouth,

genital or peri-anal areas examined)?

3.5.5

Do staff sensitively inquire about young people’s sexual

partner(s) (e.g. where the gender of partners is

not presumed, nor is the number of regular or casual

partners)?

3.5.6

Are contact cards (in the correct language) given to all

young people presenting with STIs and reasons

explained to them about the importance of all of their

sexual partner(s) coming for diagnosis/treatment?

3.5.7

Are youthful and easily understandable resource

pamphlets available in all consultation and examination

rooms on:

Signs and symptoms of common STIs

HIV and STI co-infection risks

VCT (purpose and process)

Common misconceptions about STIs

Rationale of STI treatment / management

Importance of treatment compliance and the return

visit

Importance of condom use while being treated, and

to avoid future infections

Strategies to avoid unsafe sex

3.5.8

Is the importance of abstaining from sexual intercourse

during the treatment period discussed with all young

people presenting with an STI?

3.5.9

Do clients receive written instructions about the

following:

Risks associated with the treatment they are

receiving

Warning signs of complications

Where to go for emergency and follow-up care
 3.5.10

29

**CRITERION 3.7**

**An HIV programme of care is provided.**

Is the importance of consistent condom use and the

benefits of using dual-method contraception to prevent

pregnancy and STIs/HIV stressed with all sexually

active young people?	3.5.11

Are a supply of male condoms and dildos available in all

consultation and examination rooms for demonstrations

of proper use and role plays of partner negotiation?

3.5.12

Are condoms placed in strategic places throughout the

clinic so that they can be easily accessed without seeing

a provider (and causing possible embarrassment for a

young person)?	3.5.13

**CRITERION 3.6**

**A high quality voluntary counselling and testing service is provided.**

**Questions 1st 2nd 3rd 4th**

**appraisal appraisal appraisal appraisal**

Are young people presenting with suspected

opportunistic infections routinely counselled to have

an HIV test?

3.6.1

Are young people presenting with STIs routinely

counselled to have an HIV test?

3.6.2

Are pregnant adolescents and youth routinely

counselled to have an HIV test?

3.6.3

Does pre and post test counselling take into account

the specific vulnerabilities of young people?

3.6.4

Are HIV counsellors trained on how to attend to the

needs of adolescents and youth who are infected with

HIV and/or to refer for ongoing support?

3.6.5

Are HIV test results recorded by age and gender of

the client?	3.6.6

30

**CRITERION 3.8**

**Questions 1st 2nd 3rd 4th**

**appraisal appraisal appraisal appraisal**

Are all staff aware of the factors that are driving the

high rates of HIV infection in young people?

3.7.1

Can all healthcare providers accurately assess the

prognosis of HIV or AIDS by recognizing and

diagnosing the common opportunistic infections?

3.7.2

Do staff promote healthy lifestyles and safer sexual

practices with young people who have HIV or AIDS,

including the importance of:

A positive mental attitude

Good nutrition (including vitamin and mineral

supplementation)

Maintaining healthy lifestyle choices

Limiting drug or alcohol use

Avoiding re-infection with HIV and STI by

practicing safe sex

Early treatment of illnesses

3.7.3

Is PMTCT counselling provided for all HIV-positive

adolescent / youth expectant mothers?

3.7.4

Are all healthcare providers aware of the policy

guidelines and recommendations for feeding infants of

HIV positive mothers?

3.7.5

Are all healthcare providers able to assess adolescent /

youth mothers’ circumstances and counsel her about

which feeding practices are most suitable to her

circumstances?

3.7.6

Do all young people who are HIV positive or affected by

HIV/AIDS receive counselling at the clinic?

3.7.7

Does the clinic have a link to other community-based or

government organizations that provide counselling and

social support services for adolescents with HIV or

AIDS?	3.7.8

Does the clinic have a link to community-based

organizations who provide home-based care for young

people with AIDS?	3.7.9

31

**Contraceptive information, counselling and methods are provided.**

**Questions 1st 2nd 3rd 4th**

**appraisal appraisal appraisal appraisal**

Are healthcare providers aware of protocols for

appropriate contraception for adolescents (e.g.

condoms and pills or condoms and injectable

contraceptive as opposed to IUDs)?

3.8.1

Do young people who are seeking contraceptive

services receive the following information:

How methods work and how they are used

Which methods do and do not provide protection

against STIs and HIV

Health benefits of various contraceptive methods

Common side effects of contraceptive methods

Warning signs of complications of contraceptive

various methods

How and when to obtain a re-supply of

contraceptives

Changing contraceptive methods if a method

proves to be unsuitable

Where, when and why to return for follow-up care

How to communicate with their partners about

contraception and their method of choice

How to access emergency contraception

3.8.2

Do contraceptive clients receive written information

about the following:

Risks associated with the contraceptive method

they are receiving

Warning signs of complications

Where to go for emergency and follow-up care

3.8.3

Are young people who are given oral or injectable

contraceptives routinely encouraged to practice dual-

method contraception?

3.8.4

Are condoms provided to all sexually active

adolescents and youth (including information about

proper use and counselling on partner negotiation)?

3.8.5

Is emergency contraception available to young people?	2.8.6

32

Are young people who receive emergency

contraception encouraged to test for HIV?

3.8.7

**CRITERION 3.9**

**Services are provided to adolescent girls for pregnancy.**

**Questions 1st 2nd 3rd 4th**

**appraisal appraisal appraisal appraisal**

Do healthcare providers receive training on the risks

associated with adolescent pregnancy/births?

3.9.1

Are healthcare providers able to take a history and

perform a physical examination and tests according to

protocols and guidelines for pregnant adolescents?

3.9.2

Are pregnant adolescents under the age of 18 provided

with written information on the warning signs of serious

complications that are related to their young age?

3.9.3

Are staff members able to provide education and

counselling to each pregnant adolescent, her partner

and/or family on:

Monitoring signs of problems (e.g. bleeding,

toxemia, premature labour)

Nutrition (e.g. risks associated with anaemia)

STIs / HIV

Delivery

Risks and complications associated with adolescent

pregnancy

The importance of regular antenatal care and

institutional deliveries

Processes on booking for the delivery

3.9.4

Are pregnant adolescents offered dietary supplements

as needed (e.g. iron, folic acid and iodine)?

3.9.5

Are efforts made to educate the wider community about

the risks associated with adolescent pregnancy/birth and

the measures that should be taken to support pregnant

teens (e.g. information on the importance of regular

antenatal care and institutional deliveries for

adolescents)?

3.9.6

Are all adolescent/youth mothers provided with the

following information about newborn and child care:

Newborn and infant care techniques

Newborn and infant feeding practices

33

Nutritional requirements

Immunization schedule

Growth monitoring

Hygiene and dental care

Prevention and treatment of illness (e.g. diarrhea,

acute respiratory illness)

3.9.7

Are staff members able to offer appropriate pre and post-

TOP counselling and referral for pregnant

adolescents/youth requesting termination of their

pregnancy?

3.9.8

Is there a system in place for prompt referral and

treatment of adolescents/youth presenting with abortion-

related complications?	3.9.9

**CRITERION 3.10**

**Information, counselling, and appropriate referral for violence/abuse and mental**

**health problems are provided.**

**Questions 1st 2nd 3rd 4th**

**appraisal appraisal appraisal appraisal**

Can healthcare providers identify signs and symptoms

of common mental health problems and make

appropriate referrals (e.g. depression, suicidal

tendencies, eating disorders, etc.)?	3.10.1

Can healthcare providers identify signs and symptoms

of drug or alcohol abuse and make appropriate

referrals?

3.10.2

Can healthcare providers identify signs and symptoms

of sexual abuse and make appropriate referrals?

3.10.3

Can healthcare providers identify signs and symptoms

of domestic violence and make appropriate referrals?

3.10.4

Are guidelines available on information that staff should

cover during counselling sessions with young people

who have been abused?

3.10.5

Are staff actively involved in promoting mental health,

through education on:

Substance abuse

Stress management

34

Early signs and symptoms of mental disorders

Coping with the psychological impact of physical or

sexual abuse

Identifying suicidal tendencies

3.10.6

Is there a system in place to ensure that a young

person in crisis receives care from the same

practitioner?

3.10.7

Is there a ‘victim friendly’ service area available for

young people in crisis?

3.10.8

Are there change of clothing packs available in

adolescent sizes?	3.10.9

35

**STANDARD 4**

**The clinic has a physical environment conducive to the provision of adolescent-**

**friendly health services.**

**INTENT**

**The clinic provides a safe, clean environment, including infection prevention**

**measures. The clinic ensures client privacy. An effort is made to make the clinic**

**comfortable and attractive to adolescents.**

**CRITERION 4.1**

**Consultations with adolescent clients occur in a place that assures privacy.**

**Questions 1st 2nd 3rd 4th**

**appraisal appraisal appraisal appraisal**

Do consultations take place in an area and a

manner that assures that other people cannot see or

overhear?

4.1.1

**CRITERION 4.2**

**The clinic is clean and comfortable for adolescents**

**Questions 1st 2nd 3rd 4th**

**appraisal appraisal appraisal appraisal**

Are there seats in the waiting room for each client

awaiting service?

4.2.1

Is the furniture clean, sturdy and undamaged?

4.2.2

Does the waiting room have sufficient lighting for clients

to read?

4.2.3

Is there adequate lighting in the examination and

procedure rooms?

4.2.4

Is the clinic well ventilated (e.g. fresh air, no smoking or

bad odors)?	4.2.5

36

Has an effort been made to make the clinic

environment appealing for young people (e.g. brightly

painted walls, youthful posters, décor that relates to

young people’s taste and interests, etc.)?	4.2.6

Are young people involved in providing a friendly,

welcoming environment for other youth?

4.2.7

Are all areas that clients use clean and well

maintained?

4.2.8

Are clients’ toilets available and in working condition?

4.2.9

Are clients’ toilets clean?

4.2.10

**CRITERION 4.3**

**Appropriate infection control procedures are practiced.**

**Questions 1st 2nd 3rd 4th**

**appraisal appraisal appraisal appraisal**

Are paper towels, soap, and water available for

washing hands after use of the toilet?

4.3.1

Are paper towels, disinfectant and water available for

washing hands for pre/post client contact?

4.3.2

Is safe drinking water available (e.g. chlorinated or

boiled)?

4.3.3

Is there adequate water available for cleaning and

washing equipment?

4.3.4

Is disinfectant available for sterilizing equipment?	4.3.5

37

Are infection control guidelines regularly practiced?

For example:

Wearing gloves for drawing blood

Wearing gloves for pelvic exams

Proper sharps disposal

Proper decontamination/sterilization of reusable

instruments

Proper handling of contaminated materials and

medical waste

4.3.6

Do staff wash their hands appropriately:

After arriving for and leaving work

Before and after clinical procedures

Before and after using gloves

After handling waste

After using the toilet

Before and after eating

4.3.7

Are infection control guidelines reviewed with staff at

least once a year with all staff members?

4.3.8

Are infection control guidelines reviewed with staff

during orientation?

4.3.9

Are all staff aware of universal precautions to prevent

HIV infection?

4.3.10

Is post-HIV exposure prophylaxis available for

adolescent clients and for staff members?	4.3.11

38

**STANDARD 5**

**The clinic has drugs, supplies, and equipment necessary to provide the essential**

**service package for youth-friendly healthcare.**

**INTENT**

**YFS essential services are provided with the appropriate drugs and supplies and**

**equipment.**

**CRITERION 5.1**

**Necessary drugs and contraceptives are regularly available for essential service**

**package case management.**

**Questions 1st 2nd 3rd 4th**

**appraisal appraisal appraisal appraisal**

Are the following contraceptives in stock:

Combined pills

Mini-pills

Injectables

Male condoms

Female condoms

Emergency contraceptive pills	5.1.1

Are the following drugs or equivalents in stock to provide

STI treatment:

Metronidazole

Doxycycline

Erythromycin

Ciprofloxacin

Spectinomycin /Rocephine

Benzathine benzylpenicillen

Clotrimazole/Gyno pevaryl	5.1.2

Are the following drugs or equivalents in stock to prevent

and treat opportunistic infections and other HIV related

diseases:

Trimethoprim/sulfamethoxazole/bactrim

Fluconazole/mycostatin

Rifampicin/rifafour	5.1.3

Is there a system in place for maintaining an up-to-date

drug inventory:

Stocks are secure

39

Stock cards used and up-to-date

Orders are placed regularly and on time and checked

when received against the order,

Stocks are kept orderly, with FEFO (first expiry, first

out) followed

No expired stock, the drugs ordered follow EDL

principles

Stock stored according to stipulated conditions to

preserve potency (e.g. refrigeration, protected from

light)

Stock is easily accessible

Stock is stored away from water, heat, and other

hazards	5.1.4

Have all drugs (listed above) been consistently in stock in

the past six months?

5.1.5

Is there a mechanism in place for obtaining emergency

supplies of drugs when needed?

5.1.6

Is there a suitable medicine room and medicine

cupboards that are kept locked (e.g. with burglar bars)?

5.1.7

Do healthcare providers know which medication

substitutions may be used in the event of a stock-out (e.g.

different types of pills, antibiotics for STI treatment,

contraceptive methods)?	5.1.8

**CRITERION 5.2**

**Supplies are available for YFS essential service package case management.**

**Questions 1st 2nd 3rd 4th**

**appraisal appraisal appraisal appraisal**

Are pregnancy test kits in stock?

5.2.1

Are the following forms in stock:

Risk Assessment

Client record

History taking

Referral

Contraceptive record

Patient treatment cards

Notification forms

All needed laboratory request and transfer

forms	5.2.2

40

Is there a system in place for maintaining an

inventory of supplies?

5.2.3

Are the following supplies in stock:

Needles

Syringes

Gloves

Hand-washing soap

Disposable towels

Specula

Dildo in each consultation room

Disinfectant-cleaning fluid

5.2.4

Have all supplies been in consistently in stock in

the past six months?

5.2.5

**CRITERION 5.3**

**Working equipment is available for the provision of the essential service package**

**case management.**

**Questions 1st 2nd 3rd 4th**

**appraisal appraisal appraisal appraisal**

Is the following equipment widely available?

Scales

Sphygmomanometer

Exam lights

Vaginal specula

Stethoscopes

Fetoscope with meter

Hb meter

5.3.1

Has all equipment been functioning within the last

six months?

5.3.2

Is there a system in place for procuring, maintaining

and repairing equipment?

5.3.3

Are sterile instruments stored in such a way that

they cannot be re-contaminated?	5.3.4

41

**STANDARD 6**

**Information, education, and communication promoting behaviour change and**

**consistent with the YFS essential service package is provided.**

**INTENT**

**The clinic is recognized as a resource centre and focal point for promoting healthy**

**lifestyles for adolescents. The clinic has a role and responsibility to develop**

**channels for sharing SRH information and materials within the community. The**

**materials are accurate, simple, targeted to adolescents and consistent with the YFS**

**essential service package. The materials are available in appropriate languages.**

**CRITERION 6.1**

**The clinic has accurate, easily understandable information and education materials**

**appropriate for adolescents available.**

**Questions 1st 2nd 3rd 4th**

**appraisal appraisal appraisal appraisal**

Are educational materials or other media products

related to the YFS service package available in the

waiting areas (e.g. videos with audiovisual equipment,

pamphlets, posters, etc.)?	6.1.1

Are educational materials available on at least 5 topics

related to young people’s development and healthy

lifestyle choices? For example:

Body awareness

Gender

Interpersonal relationships

Self-esteem

Negotiation skills

Assertiveness

Communication skills

Decision making

Peer pressure	6.1.2

Are educational materials available on the following

health topics?

Anatomy and physiology of the reproductive

system

Physical development during adolescence

Pregnancy prevention

Pregnancy care

Common STIs and STI prevention

STI treatment

42

**CRITERION 6.3**

HIV prevention

Living with HIV or AIDS

Substance Abuse

Sexual violence

Mental health

Nutrition	6.1.3

Are the educational materials specifically targeted to

young people?

6.1.4

Are the educational materials available in local

language(s)?

6.1.5

Are sufficient educational materials available for young

people to take home?

6.1.6

Is there a system for maintaining an inventory of

educational resources?

6.1.7

**CRITERION 6.2**

**Healthcare workers provide information and education to adolescents at the clinic**

**and in the community**

**Questions 1st 2nd 3rd 4th**

**appraisal appraisal appraisal appraisal**

Do young people receive health education while they

await service (e.g. health talks, group

discussions/debates)?

6.2.1

Do healthcare providers use youthful educational

material and/or role plays to inform young people about

relevant health issues during consultations?

6.2.2

Are healthcare providers involved in educational

activities for young people within the community (e.g.

health workshops, life skills sessions, health talks,

etc.)?	6.2.3

Do healthcare providers distribute youthful educational

material at these community events?	6.2.4

43

**Adolescents are involved in educational activities at the clinic and in the**

**community.**

**Questions 1st 2nd 3rd 4th**

**appraisal appraisal appraisal appraisal**

Do youth provide peer-education to other young people

in the clinic (e.g. while they await service or in the

chillroom)?	6.3.1

Do youth provide peer-education to other young people

in the community (e.g. at local schools or youth serving

organizations)?

6.3.2

Are peer educators being selected on the basis of

merit? For example:

Commitment to good reproductive health

Credibility as a role model, especially in

regard to behaviours the YFS advocates

Respect for peers

Ability to hold confidences

Excellent social skills (e.g. ability to interact

with both peers and adults, honesty, caring,

trustworthiness)

Excellent communication skills (e.g. ability to

speak in public and hold public’s interest,

ability to be understood)

Age, language, geographic location and

ethnicity that are similar to the target audience

6.3.3

Is there a system in place to handle turnover of peer

educators?	6.3.4

44

Are peer educators given a clear job description or key

performance areas (KPAs)? For example:

Facilitating a minimum number of peer

education sessions/month (both one-on-one

and group sessions)

Facilitating a minimum number of community

youth events/month (e.g. youth festivals,

parent-youth dialogues, youth debates, etc.)

Maintaining the upkeep of the chillroom

Attending YFS Team meetings

Participation in YFS service improvement

initiatives

Keeping up-to-date records

Keeping up-to-date on topics covered in

sessions (via self study or participation in

trainings/conferences)

Providing referrals

Mentoring other peer educators

Distributing educational materials and

condoms

6.3.5

Has a member of staff been designated as the line

manager of the peer education programme?

6.3.6

Are peer educators well trained to carry out each of

their roles (e.g. YFS spokesperson, peer educator, peer

counsellor, event coordinator, etc.)?

6.3.7

Is there a system in place to regularly monitor and

evaluate peer educators’ performance?	6.3.8

Are peer educators given regular constructive feedback

on how they can improve their performance?	6.3.9

Are any tangible incentives provided to peer educators

(e.g. stipend, reimbursement for transportation, food,

training, academic credit, performance bonuses, etc.)?

6.3.10

Are any intangible incentives provided to peer

educators? (e.g. building a resume, involvement in

formulating clinic policy, acquaintance with important

community figures, etc.)	6.3.11

45

**STANDARD 7**

**Systems are in place to train staff to provide effective adolescent-friendly health**

**services.**

**INTENT**

**A system is in place to identify staff learning needs and to develop plans to meet**

**these needs. Training and development activities are conducted to prepare staff to**

**effectively carry out youth friendly services. Staff have conducive attitudes toward**

**working effectively with young people.**

**CRITERION 7.1**

**The clinic has a training plan that ensures that all staff effectively provide the YFS**

**essential service package.**

**Questions 1st 2nd 3rd 4th**

**appraisal appraisal appraisal appraisal**

Has a staff training needs assessment been

conducted in relation to delivery of YFS?

7.1.1

Has a YFS training plan been developed?

7.1.2

Is the YFS training plan updated periodically (e.g.

annually)?

7.1.3

**CRITERION 7.2**

**Staff are well trained to provide the YFS essential service package, using the**

**standard case management guidelines and other related guidelines/protocols.**

**Questions 1st 2nd 3rd 4th**

**appraisal appraisal appraisal appraisal**

Have healthcare providers been trained in the

following:

Syndromic management of STIs

Voluntary counselling and testing for adolescent

clients

HIV programme of care for adolescent clients

Contraceptive information, counselling and

provision of contraceptive methods

Pregnancy testing and counselling

Ante-natal and postnatal care for adolescent

46

clients

Pre and post-TOP counselling and referral for

adolescent clients

Primary healthcare management of violence;

sexual abuse; mental health problems

(grief/depression/suicide; drugs; alcohol abuse)

7.2.1

Is a record kept of all training activities attended by

each staff member?

7.2.2

Is there a system in place to assist staff to transfer

their learning into regular practice?

7.2.3

Has the clinic collaborated with other training

organizations for further staff development related to

YFS?	7.2.4

**CRITERION 7.3**

**Staff are trained and developed to assist and serve youth in a non-judgmental,**

**supportive manner.**

**Questions 1st 2nd 3rd 4th**

**appraisal appraisal appraisal appraisal**

Are activities conducted to prepare staff to perform

their responsibilities as ‘youth friendly’ providers? For

example, are staff:

Knowledgeable of young people’s needs and

vulnerabilities

Friendly and responsive to young people

Trustworthy

Good communicators with young people

7.3.1

Do staff performance reviews include feedback

regarding their interaction with adolescent and youth

clients?	7.3.2

47

**STANDARD 8**

**Adolescents receive an adequate psycho-social and physical assessments.**

**INTENT**

**An adequate and appropriate assessment is conducted that takes into account the**

**social, economic and cultural background of the adolescent, as well as their risk for**

**HIV, STIs and unintended pregnancies.**

**CRITERION 8.1**

**Healthcare providers take an appropriate history**

**Questions 1st 2nd 3rd 4th**

**appraisal appraisal appraisal appraisal**

Do providers document comprehensive patient

histories:

Age

Sex

Presenting complaints

Past medical problems

Family medical history

O&G history (LMP, pregnancies, TOPs,

births)

Sexual history

Social history (home and social

circumstances)

Risk behaviours (smoking, drinking, drugs,

sexual partners, use of condoms)

8.1.1

**CRITERION 8.2**

**Healthcare providers perform appropriate physical examinations and investigations**

**according to standard case management guidelines/protocols.**

**Questions 1st 2nd 3rd 4th**

**appraisal appraisal appraisal appraisal**

Does the assessment/examination specifically address

the presenting complaint?

8.2.1

Does the assessment/examination take young people’s

psycho-social status into account?	8.2.2

48

Does the assessment/examination take the young

person’s risk profile into account?

8.2.3

Do the healthcare providers perform examinations

according to standard guidelines/protocols?

8.2.4

Are investigations carried out as indicated in the case

management guidelines?

8.2.5

Do healthcare providers give adequate explanation of

the process if an examination or procedure is required to

alleviate young people’s fears (e.g. what will be done,

what to expect, why it is needed)?	8.2.6

**CRITERION 8.3**

**Assessments are undertaken with consideration to the comfort, dignity, and**

**modesty of the adolescent**

**Questions 1st 2nd 3rd 4th**

**appraisal appraisal appraisal appraisal**

Do healthcare providers perform physical examinations

and other procedures with the young person’s dignity,

modesty and comfort in mind (e.g. client is only

exposed when necessary)?	8.3.1

**CRITERION 8.4**

**Healthcare providers ensure that no opportunity is missed to comprehensively**

**assess adolescent health needs and risks.**

**Questions 1st 2nd 3rd 4th**

**appraisal appraisal appraisal appraisal**

Is a behaviour-risk assessment completed for each

adolescent during their first visit and updated regularly

(e.g. annually)?

8.4.1

Is a social history completed for each adolescent

during their first visit and updated regularly (e.g.

annually)?

8.4.2

Do clients receive counselling on issues related to

their presenting complaint?	8.4.3

49

Are opportunities for health promotion taken during all

consultations (e.g. encouragement of dual protection

for a young person seeking oral contraception)?	8.4.4

50

**CRITERION 9.2**

**Adolescents are encouraged to express their concerns, ask questions, and discuss**

**STANDARD 9**

**Adolescents receive individualized care based on standard case management**

**guidelines/protocols.**

**INTENT**

**Adolescents are cared for using standard case management guidelines. Service**

**delivery guidelines are available for the YFS essential service package.**

**Communication with adolescents is conducted in a way that encourages them to**

**participate in decisions about their care.**

**CRITERION 9.1**

**Service delivery guidelines for the essential service package are available and**

**regularly referred to.**

**Questions 1st 2nd 3rd 4th**

**appraisal appraisal appraisal appraisal**

Are up-to-date treatment guidelines/protocols and drug

lists available for the following?

Oral contraceptive pills

Emergency contraception

Injectables

Condoms

Pregnancy counselling and testing

Antenatal and postnatal care

Pre and post-TOP counselling and referral

Syndromic management of STIs

HIV pre and post testing counselling

Primary healthcare management of: violence;

sexual abuse; mental health problems

(grief/depression/suicide; drugs; alcohol abuse)	9.1.1

Is care and treatment provided according to standard

disease management protocols and standard

treatment guidelines (STGs)?	9.1.2

Is clinical practice routinely monitored to ensure that

services are being delivered according to the protocols

/ STGs?	9.1.3

51

**their treatment options.**

**Questions 1st 2nd 3rd 4th**

**appraisal appraisal appraisal appraisal**

Do healthcare providers actively encourage young

people to express their concerns and ask questions

about their treatment?	9.2.1

Are the risks, benefits, and potential complications of

treatments and procedures discussed with young

people using simple terminology?	9.2.2

Do healthcare providers avoid influencing young

people’s decisions about treatment by telling them

‘what is best for them’?	9.2.3

**CRITERION 9.3**

**Healthcare providers use effective counselling skills during consultations.**

**Questions 1st 2nd 3rd 4th**

**appraisal appraisal appraisal appraisal**

Do staff respect young people’s opinions and choices,

even when they are not the same as their own?	9.3.1

Have staff been trained on how to effectively counsel

adolescent/youth clients (e.g. using positive body

language, verbal encouragement, paraphrasing,

sensitive approaches for answering difficult questions,

etc.)?	9.3.2

52

**STANDARD 10**

**The clinic provides continuity of care for adolescents.**

**INTENT**

**Systems, procedures, and records are maintained in a way to promote effective**

**follow up care. Effective referrals and counter-referrals are made. Adequate and**

**clear information is given to facilitate the adolescent’s return and ongoing use of**

**services.**

**CRITERION 10.1**

**Adolescents are given clear and understandable follow-up information**

**Questions 1st 2nd 3rd 4th**

**appraisal appraisal appraisal appraisal**

Are young people told when and why they need to

return for follow-up?	10.1.1

Are young people told to return if they experience

specific side effects or complications?	10.1.2

Do young people receive written instructions about the

following:

Risks associated with the medication they are

receiving

Warning signs of complications

Where to go for follow-up care

Where to go for emergency care

10.1.3

Do healthcare providers check to see whether the

young person has fully understood the information

given by asking him/her to repeat the instructions in

their own words?	10.1.4

**CRITERION 10.2**

**An adequate referral system for adolescent healthcare exists.**

**Questions 1st 2nd 3rd 4th**

**appraisal appraisal appraisal appraisal**

53

Are referral arrangements in place for the following

services :

Voluntary counselling and testing for HIV (if not

available on-site)

TOP

Recurrent STIs

Maternity

Drug/alcohol abuse

Mental health problems

Rape/sexual assault

Physical abuse

Social problems

Other problems that require specialized care

10.2.1

Is there a referral resource list for each type of service

mentioned above?

10.2.2

Do all staff members know which services are available

by referral to another facility, where the facility is located

and how clients can get there?	10.2.3

Have referral procedures been put in place to ensure

proper back-referrals (e.g. letter sent with patient to

referral facility, back-referral to the clinic with clear

instructions about follow-up care and medication needed

for continued care)?	10.2.4

Does the referral letter specify the following information:

Age and sex of client

Reason(s) for referral

Specific findings

Diagnosis and treatment given

Expectations for client care / support	10.2.5

Do staff take measures to ensure that the clients they

have referred to another department or facility for

services actually receive the care for which they were

referred?	10.2.6

Are referral facilities well informed about the clinic’s YFS

programme?

10.2.7

19

54

**Tool # 2**

**YFS APPRAISAL REPORT**

**Facility Name: Assessment Team Members:**

**Date:**

**YFS Coordinator:**

**SCORING KEY**

**Criterion Fully Met = 2 Points**

**Criterion Partially Met = 1 Point**

**Criterion Not Met = 0 Points**

**SCORE NOTEWORTHY FINDINGS**

**/12**

**%**

**/10**

**STANDARD/**

**CRITERIA**

**STANDARD 1**

**1.1**

**1.2**

**1.3**

**1.4**

**1.5**

**1.6**

**Points**

**% STD1**

**STANDARD 2**

**2.1**

**2.2**

**2.3**

**2.4**

**2.5**

**Points**

55

**% STD2 %**

**STANDARD 3**

**3.1**

**3.2**

**3.3**

**3.4**

**3.5**

**3.6**

**3.7**

**3.8**

**3.9**

**3.10**

**Points /20**

**% STD3 %**

**STANDARD 4**

**4.1**

**4.2**

**4.3**

**Points /6**

**% STD4 %**

**STANDARD 5**

**5.1**

**5.2**

**5.3**

**Points /6**

**% STD5 %**

**STANDARD 6**

**6.1**

**6.2**

**6.3**

56

**Points /6**

**% STD6 %**

**STANDARD 7**

**7.1**

**7.2**

**7.3**

**Points /6**

**% STD7 %**

**STANDARD 8**

**8.1**

**8.2**

**8.3**

**8.4**

**Points /8**

**%STD8 %**

**STANDARD 9**

**9.1**

**9.2**

**9.3**

**Points /6**

**%STD9 %**

**STANDARD 10**

**10.1**

**10.2**

**Points /4**

**%STD10 %**

57

**OVERALL**

**SCORE**

**____ /**

**84 points**

**= ____%**

**KEY ISSUES TO CONSIDER FOR THE WAY FORWARD:**

58

**Selected content for this chapter was adapted from:**

1. Going for Gold – A Clinic Guide to the National Adolescent Friendly Clinic Initiative. 2004. Reproductive Health

Research Unit (as part of loveLife).

2. Judith Senderowitz, Gwyn Hainsworth and Cathy Solter. A Rapid Assessment of Youth Friendly Reproductive Health

Services. 2003. Pathfinder International.

3. Judith Senderowitz, Gwyn Hainsworth and Cathy Solter. Clinic Assessment of Youth Friendly Services: A Tool for

Assessing and Improving Reproductive Health Services for Youth. 2002. Pathfinder International.

4. The Clinic Supervisors Manual (Version 1.1). 2004. The South African Department of Health; Gauteng Province.

5. The Primary Healthcare Package for South Africa – a set of norms and standards. 2000. The South African

Department of Health.
